# Supplementary material for: Time-resolved structure-function coupling in brain networks
Source: Commun Biol. 2022 Jun 2;5:532. doi: 10.1038/s42003-022-03466-x (PMC9163085; doi:10.1038/s42003-022-03466-x)
Supplement: Supplementary file 2 — Supplementary Information [file 42003_2022_3466_MOESM2_ESM.pdf]

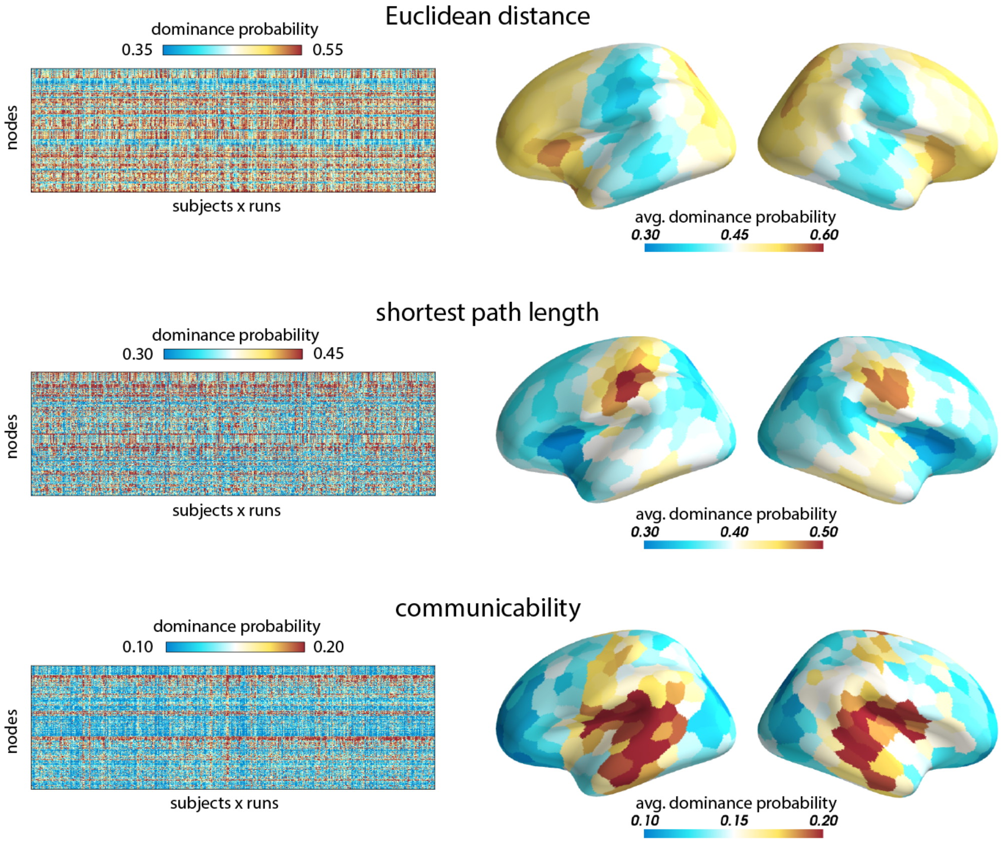

Figure S1 | **Predictor contributions.** Dominance Analysis was used to quantify the distinct contributions of the three predictors in the mulitlinear model (Euclidean distance, path length and communicability) [6, 13] (https://github.com/dominance-analysis/dominance-analysis). The technique estimates the relative importance of predictors by constructing all possible combinations of predictors and quantifying the relative contribution of each predictor as additional variance explained (i.e. gain in R^2) by adding that predictor to the models. The incremental R^2 contribution of each predictor to a given subset model of all the other predictors is then calculated as the increase in R^2 due to the addition of that predictor to the regression model. Left: dominance over time for the three predictors. Each column in the matrix shows the frequency of the predictor being the dominant predictor through time. Right: brain map of the matrix averaged over subjects and runs.

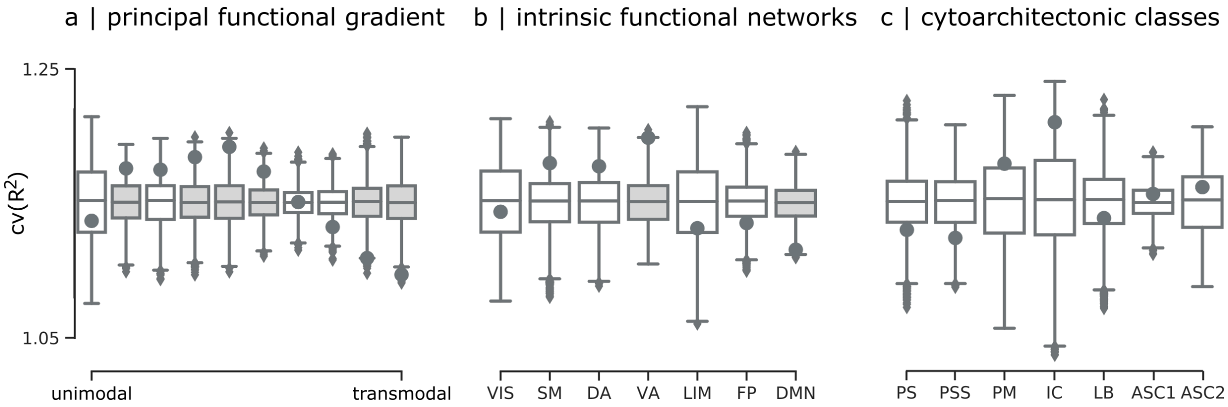

Figure S2 | **Spin test of the coefficient of variation of the structure-function coupling**. Black points show partition-specific mean $\mathrm{cv}(R^2)$ (same as results in Fig. 3c-e). Boxplots show distributions of partition-specific mean $\mathrm{cv}(R^2)$ for 10,000 spatial autocorrelation-preserving null models ("spin tests") [1, 53]. Darker (filled) and lighter (non-filled) boxes show partitions for which $\mathrm{cv}(R^2)$ is statistically significant ($p < 0.05$) and non-significant ($p > 0.05$), respectively.

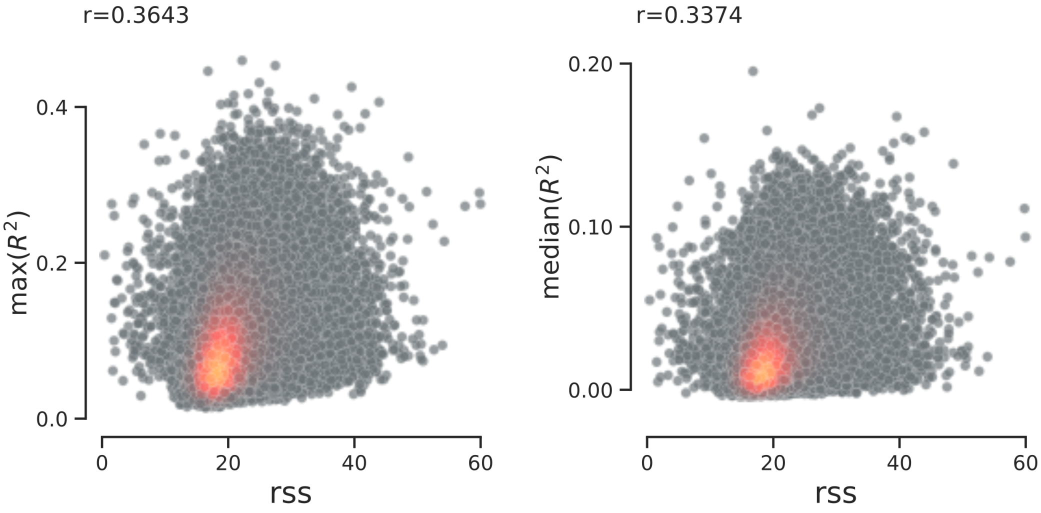

Figure S3 | **Co-fluctuation amplitude and structure-function coupling** To characterize the amplitude of the co-fluctuation time series, Esfahlani et al. calculated the root sum square (RSS) of the co-fluctuation time series across the network at each time point. The maximum and median of each of the R_t^2 vectors (column vector at figure 1c) is positively correlated with the RSS.
